# Molecular characterization of protein kinase C delta (PKCδ)-Smac interactions

**DOI:** 10.1186/s12858-016-0065-x

**Published:** 2016-05-23

**Authors:** Christian Holmgren, Louise Cornmark, Gry Kalstad Lønne, Katarzyna Chmielarska Masoumi, Christer Larsson

**Affiliations:** Lund University, Translational Cancer Research, Medicon Village, Building 404:C3, SE-22363 Lund, Sweden

**Keywords:** Protein kinase C, Smac, Protein interaction, Co-immunoprecipitation

## Abstract

**Background:**

Protein kinase C δ (PKCδ) is known to be an important regulator of apoptosis, having mainly pro- but also anti-apoptotic effects depending on context. In a previous study, we found that PKCδ interacts with the pro-apoptotic protein Smac. Smac facilitates apoptosis by suppressing inhibitor of apoptosis proteins (IAPs). We previously established that the PKCδ-Smac complex dissociates during induction of apoptosis indicating a functional importance. Because the knowledge on the molecular determinants of the interaction is limited, we aimed at characterizing the interactions between PKCδ and Smac.

**Results:**

We found that PKCδ binds directly to Smac through its regulatory domain. The interaction is enhanced by the PKC activator TPA and seems to be independent of PKCδ catalytic activity since the PKC kinase inhibitor GF109203X did not inhibit the interaction. In addition, we found that C1 and C2 domains from several PKC isoforms have Smac-binding capacity.

**Conclusions:**

Our data demonstrate that the Smac-PKCδ interaction is direct and that it is facilitated by an open conformation of PKCδ. The binding is mediated via the PKCδ regulatory domain and both the C1 and C2 domains have Smac-binding capacity. With this study we thereby provide molecular information on an interaction between two apoptosis-regulating proteins.

## Background

Apoptosis is a form of programmed cell death that was first described as a process of cellular turnover important for tissue homeostasis under physiologic conditions [1]. It also acts as a barrier to cancer development and dysregulation of apoptosis is a hallmark of most, if not all, cancer types [2]. Regulation of apoptosis is complex and involves multiple signaling pathways and proteins that are commonly grouped into two processes, the extrinsic apoptotic pathway that is activated by ligand-receptor interactions at the cell surface and the intrinsic apoptotic pathway that is activated by permeabilization of the mitochondrial membrane. Both pathways lead to activation of caspases, a group of proteins that are effectors of apoptosis [3].

When mitochondria are permeabilized during intrinsic apoptosis, proteins that participate in stimulation of apoptosis are released from the mitochondrial intermembrane space. One of the proteins released is Smac. Smac is a 25 kDa protein requiring posttranslational modification for maturation and activation. After translation, a mitochondrial targeting signal (MTS) located in the N-terminal part of the protein directs it to mitochondria and upon entry, the MTS is cleaved off yielding mature Smac [4]. When released from mitochondria, mature Smac facilitates apoptosis by binding to and inhibiting proteins of the inhibitor of apoptosis protein (IAP) family such as X-linked inhibitor of apoptosis protein (XIAP) and cellular inhibitor of apoptosis protein (cIAP) 1/2, which leads to disinhibition of caspases [4–6] and redirection of TNFα-signaling towards caspase-8 activation [7, 8]. Besides protein localization, Smac has been reported to be a target of several kinases that act to regulate its apoptotic functions [9–11]. Furthermore, Smac may have additional apoptotic functions that are independent of its ability to bind IAPs [12].

The apoptotic pathways are influenced by numerous signaling proteins and among them is the protein kinase C (PKC) family of proteins. Several PKC isoforms have been linked to regulation of apoptosis. One of these isoforms is PKCδ, a protein known to be an important regulator of apoptosis with mainly pro-apoptotic functions [13, 14]. However, PKCδ has several anti-apoptotic functions as well which has been described in a previous study in our group as well as in several other publications [15–18].

In a previous study we found that PKCδ and Smac interact in breast cancer cell lines and that the interaction is disrupted during paclitaxel-mediated cell death [19]. Since the interaction between the two proteins appears to have a role in cell death, we aimed at characterizing the molecular determinants of the interaction. In this study, we show that PKCδ and Smac bind directly to each other via the regulatory domain of PKCδ. We also show that the binding between the proteins is stimulated when PKCδ is in an open conformation.

## Results

### PKCδ and Smac bind directly to each other and the binding is stimulated when PKCδ is in an open conformation

**Fig. 1:**
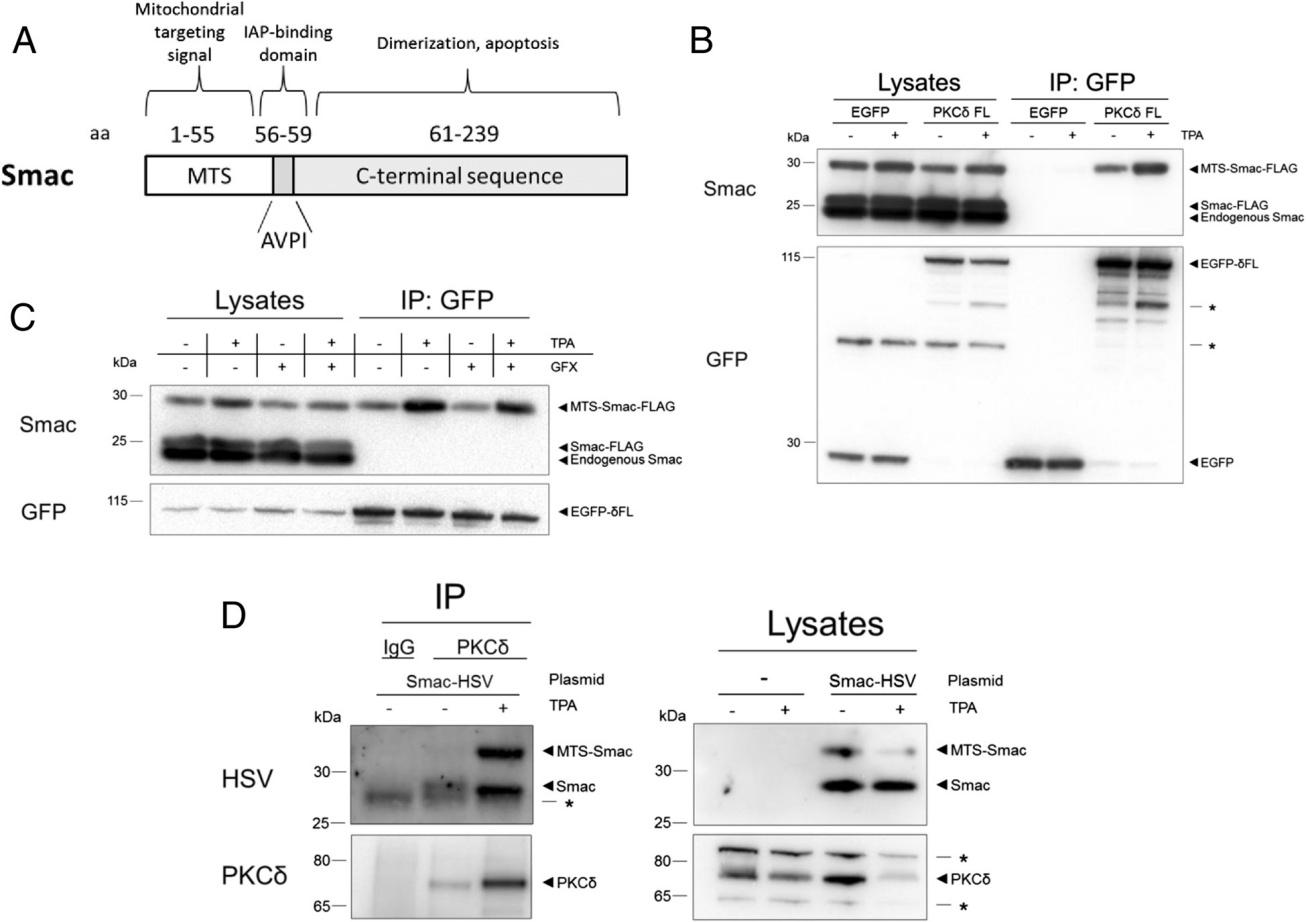
PKC activation enhances the interaction between PKCδ and Smac in cells. **a** Schematic of Smac structure. **b**, **c** COS-7 cells were transfected with vectors encoding EGFP-tagged full-length PKCδ and Smac-FLAG. After transfection, indicated cells were treated with 16 nM TPA (**b**) with or without 2 μM GF109203X (**c**) for 16 h. Lysates were then used for GFP-immunoprecipitation. **d** MCF-7 cells were transfected with a vector encoding Smac-HSV. After transfection, indicated cells were treated with 16 nM TPA for 16 h. Lysates were then used for PKCδ-immunoprecipitation. Figures are representatives from three experiments. Asterisk indicates non-specific band

In order to analyze the effect of PKC activation and inhibition on the interaction with Smac, COS-7 cells were transfected with tagged PKCδ- and Smac-constructs followed by co-immunoprecipitation. We observed that PKCδ primarily interacts with a larger Smac variant, conceivably corresponding to Smac with an intact MTS (Fig. 1a and b), and this interaction was further enhanced by treatment with the PKC activator TPA (Fig. 1b and c). However, treatment with the PKC inhibitor GF109203X influenced neither the basal nor the TPA-facilitated PKCδ-Smac interaction (Fig. 1c).

A similar effect of TPA was observed by immunoprecipitation of endogenous PKCδ from MCF-7 breast cancer cells expressing a HSV-tagged Smac (Fig. 1d). While overexpressed PKCδ in COS-7 cells preferentially co-precipitated immature Smac harboring an intact MTS, endogenous PKCδ preferentially co-precipitated mature Smac in MCF-7 cells. In this setting, TPA treatment enabled interaction with the immature, MTS-containing form (Fig. 1d).

**Fig. 2:**
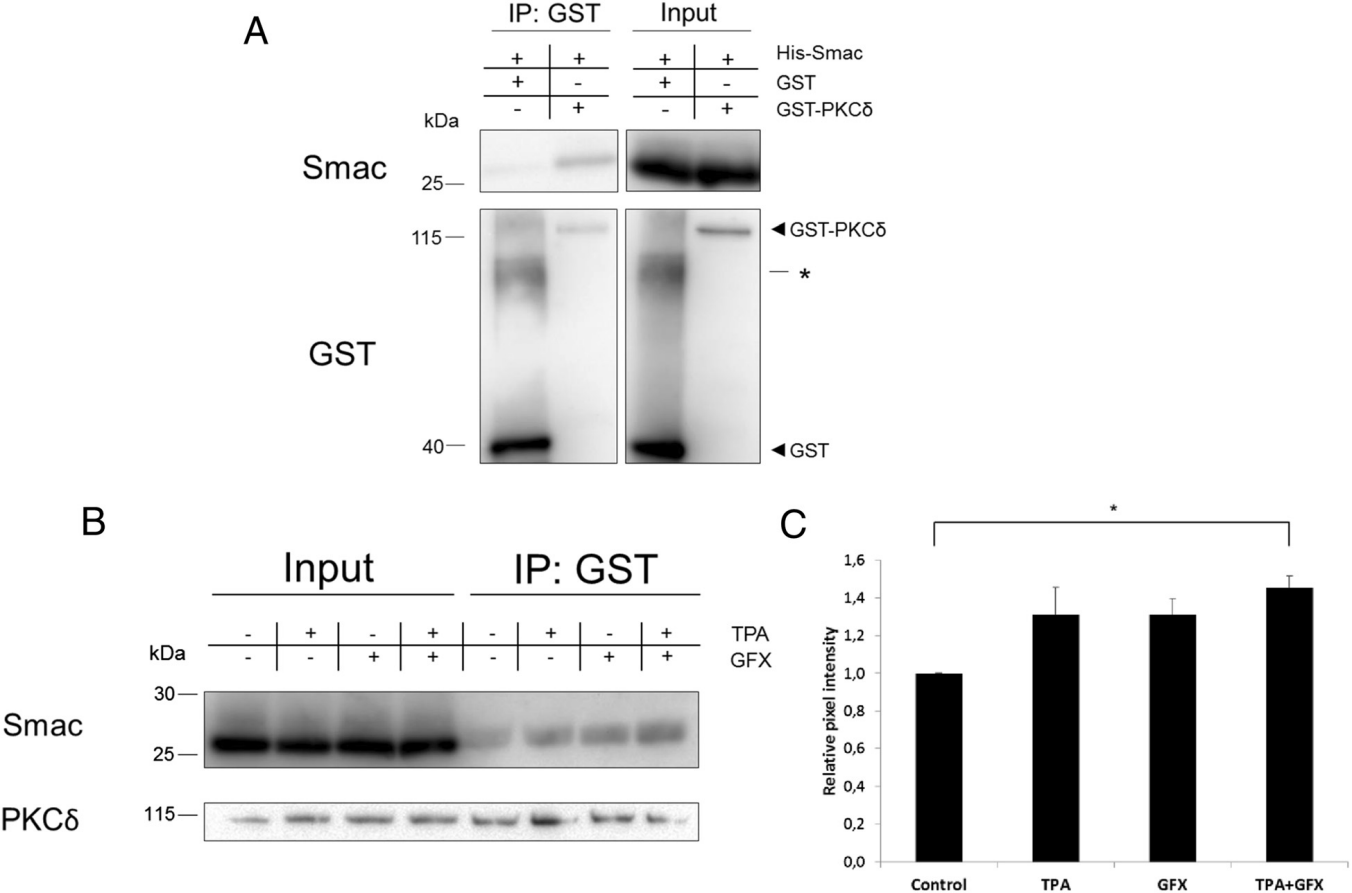
PKCδ and Smac bind directly to each other. **a**–**c** His-Smac was incubated with GST-PKCδ or GST for 1 h before GST-immunoprecipitation. In **b**, His-Smac was incubated with GST-PKCδ for all samples. Fractions were collected and analyzed with Western blot. 20 nM TPA and/or 2 μM GF109203X (GFX) were included in the binding reactions where indicated. Figures are representatives from three experiments. Asterisk indicates non-specific band. **c** Quantification of signal intensity of Smac bands in the IP-fractions from (**b**). Band intensities were normalized to input fractions and control. Data represents mean ± SEM from three independent experiments. * indicates *p* < 0.05

To investigate if PKCδ and Smac bind directly to each other, purified recombinant His-tagged Smac and GST-tagged PKCδ were incubated in a binding reaction followed by GST-immunoprecipitation. We found that His-Smac was co-precipitated with GST-PKCδ whereas almost no co-precipitation was seen with GST alone, showing that the proteins can directly bind each other (Fig. 2a). To investigate if the activation status of PKCδ affects its interaction with Smac also in this setting, TPA and/or GF109203X were added to the binding reaction. Both TPA and GF109203X, alone and in combination, seemed to yield an increased binding. However, upon quantification only the combination gave a significant increase (Fig. 2b and c). Since GF109203X inhibits PKC activity but also stabilizes the open, active conformation of PKCs [20], the results indicate that the Smac-PKCδ interaction is facilitated when PKCδ is in an open conformation, independent of PKCδ activity.

### Smac binds to the regulatory domain of PKCδ

**Fig. 3:**
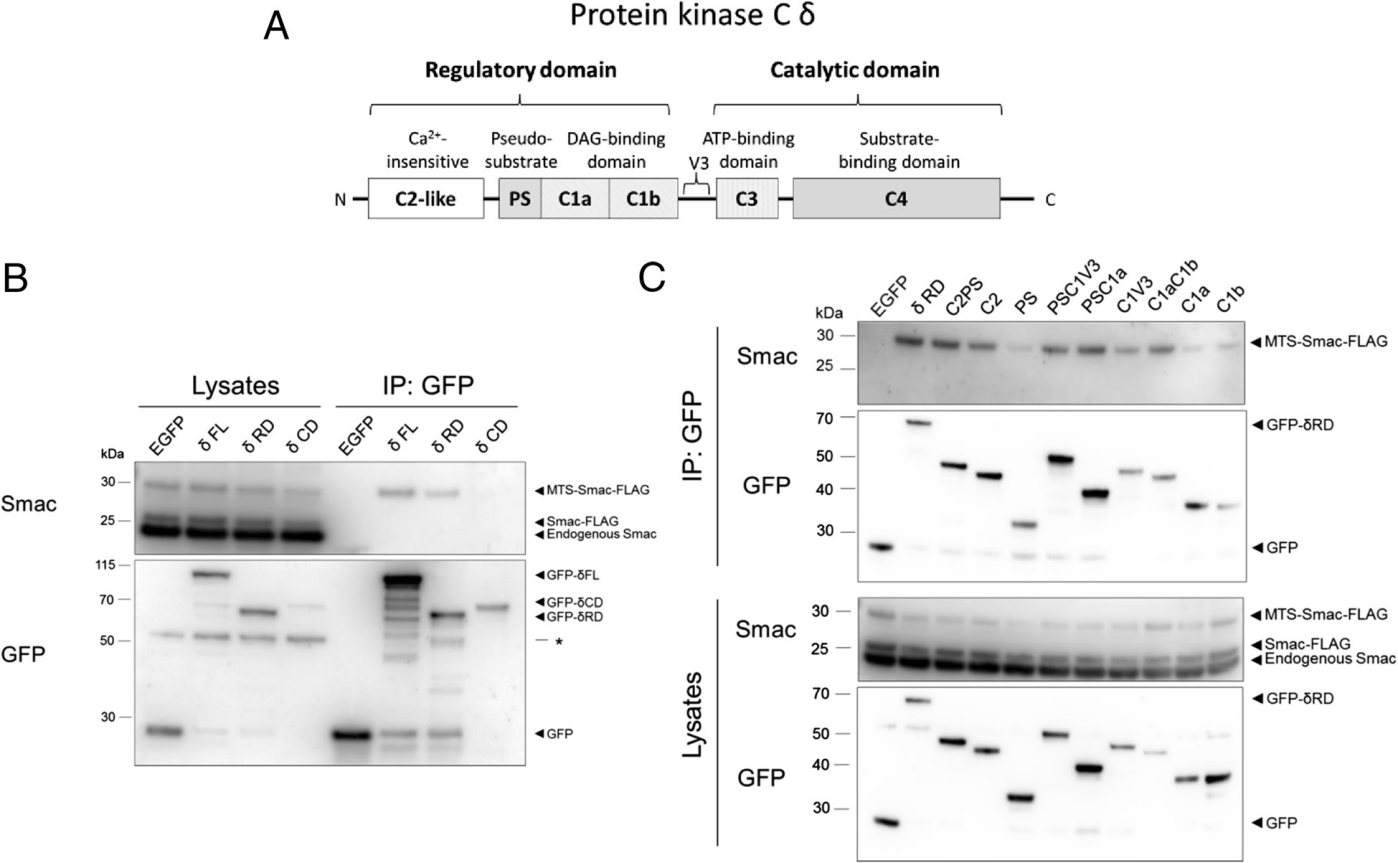
Smac binds to the regulatory domain of PKCδ. **a** Schematic of PKCδ structure. **b**, **c** COS-7 cells were transfected with vectors encoding full-length Smac-FLAG together with vectors encoding EGFP-fusions of full-length PKCδ, the regulatory domain or the catalytical domain (**b**) or different combinations of regulatory domain subregions (**c**). Lysates were then used for GFP-immunoprecipitation. FL = Full-length, RD = Regulatory domain, CD = Catalytical domain, PS = Pseudosubstrate domain. Figures are representatives from three experiments. Asterisk indicates non-specific band

We have previously reported that interaction with PKCδ is dependent on the IAP-binding domain of Smac [19]. Therefore, we focused on identifying the specific domains of PKCδ necessary for mediating the interaction. The PKCδ protein consists of multiple domains that can be grouped into an N-terminal regulatory domain and a C-terminal catalytic domain (Fig. 3a). To narrow down which parts of PKCδ that mediate the binding to Smac, co-immunoprecipitation was performed on COS-7 cells transfected with FLAG-tagged full-length Smac together with GFP-tagged full-length PKCδ or the isolated regulatory or catalytic domains. Smac co-precipitated with full-length PKCδ and the regulatory domain but not with the catalytic domain of PKCδ (Fig. 3b). We proceeded with analyzing which parts of the regulatory domain that could co-precipitate Smac in our assay. Constructs containing either the C1 or the C2 domains had the highest capacity to co-precipitate Smac (Fig. 3c). However, isolated C1 or pseudosubstrate domains did not interact with Smac to a large extent. Altogether, the results indicate that there is more than one site in the regulatory domain of PKCδ that have the ability to bind Smac.

### Smac can interact with several PKC-isoforms

**Fig. 4:**
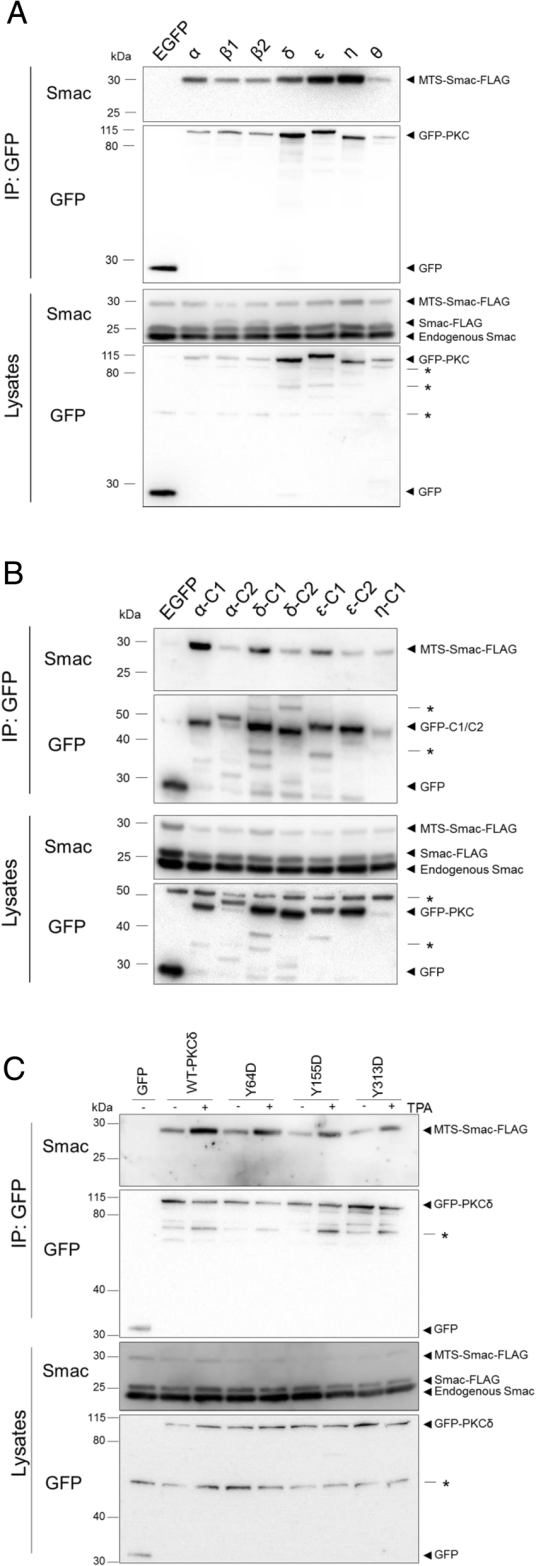
Smac can interact with several PKC-isoforms. **a**, **b**, **c** COS-7 cells were transfected with vectors encoding full-length Smac-FLAG and EGFP-fusions of full-length PKC isoforms (**a**), C1/C2 domains from different isoforms (**b**) or mutated full-length PKCδ variants carrying a tyrosine-to-aspartate mutation on Tyr-64 (Y64D), Tyr-155 (Y155D) or Tyr-313 (Y313D) (**c**). After transfection, indicated cells were treated with 16 nM TPA. Lysates were then used for GFP-immunoprecipitation. Figures are representatives from three experiments. Asterisk indicates non-specific band

Since PKC isoforms have a large degree of homology among its family members [21], we wanted to examine if other PKC family members have the ability to interact with Smac. To investigate this, COS-7 cells were transfected with vectors encoding tagged, full length versions of different PKC family members and Smac which was followed by co-immunoprecipitation. The extent to which Smac was co-precipitated varied between PKC family members. However, all PKC family members included were capable of co-precipitating Smac to some extent (Fig. 4a). We next analyzed if the tandem C1aC1b domain or the C2 domain from these isoforms was sufficient for interaction with Smac. All tested constructs could co-precipitate Smac but in general, the strongest associations were seen with the tandem C1aC1b constructs (Fig. 4b). This indicates that Smac has the ability to interact with several PKC isoforms besides PKCδ.

Amongst the PKC family members, PKCδ is known to be the most extensively tyrosine-phosphorylated isoform [22]. The importance of tyrosine phosphorylation in regulation of PKCδ functions has been shown in several publications [16, 22, 23]. Because of this, we investigated if phosphorylation on some key tyrosine residues on PKCδ could alter the association with Smac. To pursue this, vectors encoding phosphomimetic mutants of PKCδ were generated, having a tyrosine residue mutated to aspartate to mimic the negative charge imparted by phosphorylation. The vectors generated encoded Y64D, Y155D or Y313D point mutations and these residues were tested because they have been shown to induce changes in substrate binding upon phosphorylation [23–25]. The vectors were transfected into COS-7 cells together with tagged, full-length Smac. None of the phosphomimetic PKCδ mutants generated showed any change in Smac-binding compared to wild-type PKCδ under neither basal nor TPA-stimulated conditions (Fig. 4c). The results suggest that constitutive mono-phosphorylation on any of the tyrosine residues tested does not influence the binding between PKCδ and Smac.

## Discussion

In this study, we have characterized the interaction between PKCδ and Smac and found that the two proteins bind directly to each other. Furthermore, the results indicate that the interaction is facilitated by an open conformation of PKCδ.

Our data showed that TPA, a PKC activator, stimulates the interaction in both MCF-7 and COS-7 cells. The PKC inhibitor GF109203X did not suppress the interaction and it potentiated the effect of TPA on recombinant proteins. GF109203X is an inhibitor described to inhibit PKC activity by stabilizing an active, open conformation [20]. When TPA binds to the C1 domain, a hydrophobic surface is formed over the C1 domain [26], which is a key step in promoting an open PKC conformation. Therefore, our results point to a model in which the interaction between PKCδ and Smac is independent of kinase activity but is facilitated by an open conformation.

The significance of the Smac-PKCδ interaction remains to be fully understood. Since the interaction takes place via the regulatory domain of PKCδ, it is less likely that it directly blocks the catalytic site of PKCδ. A clue to the function of the interaction can perhaps instead be gained from the observation that it is the tandem C1aC1b domain and the C2 domain of PKCδ that seem to co-precipitate Smac the most. These domains regulate PKC activity in part by binding co-factors required for activation. C1 domains bind diacylglycerol whereas C2 domains bind calcium ions, both of which serve as activators of PKC function [13]. However, PKCδ lacks a classical C2 domain and instead has a C2-like domain that does not bind calcium ions, rendering the protein calcium-independent [22]. Since Smac interacts the most with these domains, it is possible that the interaction has a regulatory effect on PKCδ activity. Because our results point to a model in which an open PKCδ conformation facilitates the interaction, it can be speculated that the interaction could modulate the activity of enzymatically active PKCδ, either by stabilizing the open conformation or by inhibiting its catalytic function through allosteric regulation.

Through our studies, we found that all PKC family members that were tested had the ability to co-precipitate Smac and that both the tandem C1aC1b- and the C2-domains of different isoforms contain structures that can interact with Smac. In contrast to this, no interaction with Smac was found when endogenous PKCα or PKCε was precipitated in the MDA-MB-231 breast cancer cell line [19]. This suggests that although PKCδ is the only PKC family member described to interact with Smac so far, other PKCs seem to have the ability to interact with Smac but may not do so under endogenous conditions. One potential explanation for this is that the levels of the respective PKC family members are higher upon overexpression than in the endogenous settings tested in the previous study. This may result in the appearance of less specific interactions. Localization of proteins could also potentially explain why an interaction is only seen with PKCδ under endogenous conditions. Smac is located in mitochondria under non-stimulated conditions and in previous studies, it has been reported that PKCδ can translocate to mitochondria and interact with mitochondrial proteins [4, 15, 27, 28]. Previously, we have shown that the Smac-PKCδ interaction takes place in a mitochondria-rich fraction and not in the cytosol [19]. If the other PKC isoforms are present in lower amounts in mitochondria compared to PKCδ, this could potentially explain this preference of interaction under endogenous conditions. However, it cannot be excluded that Smac may interact with other PKC isoforms in other cell types.

We noted that PKCδ could co-precipitate both mature Smac as well as the immature pro-form carrying a mitochondrial targeting signal. In COS-7 cells, PKCδ preferentially co-precipitated the immature form whereas the opposite was observed in MCF-7 cells under non-stimulated conditions. This could potentially be explained by our approach in which we performed the co-immunoprecipitation with endogenous PKCδ in MCF-7 cells whereas in COS-7 cells, both proteins where exogenously expressed through plasmid transfections. It could be that overexpression of PKCδ in COS-7 cells causes the protein to accumulate in the cytoplasm, stimulating interaction with Smac prior to mitochondrial import and maturation.

Our studies on phosphomimetic mutants showed that substitution of tyrosine to aspartate, a negatively charged amino acid, did not change the binding affinity of PKCδ to Smac. Aspartate mimics the charge but not fully the structure of phosphotyrosine and does therefore not completely replicate a phosphorylated residue. Phosphorylations on the sites tested have previously been reported to modify the function and/or activity of PKCδ [23–25]. Since tyrosine phosphorylation is more extensive on PKCδ than on other PKC family members, we hypothesized that phosphorylation could be an explanation as to why we have been unable to detect endogenous Smac interaction with other PKC family members. The lack of visible differences on the PKCδ-Smac interaction in our studies on the phosphomimetic mutants do not support a hypothesis that the interaction is influenced by phosphorylation on the residues tested in our study.

## Conclusions

Our data demonstrate that the two apoptosis-regulating proteins Smac and PKCδ bind directly to each other. The interaction is mediated via the regulatory domain of PKCδ and the C1 and C2 domains of several PKC isoforms have Smac-binding capacity. The binding is facilitated by exposure of the regulatory domain of PKCδ and thus on an open conformation of the protein.

## Methods

### Plasmids

The plasmid vectors encoding Smac-HSV and Smac-FLAG have previously been described [4, 29]. Vectors encoding full-length EGFP-tagged PKCα, βI, βII, δ, ε, η and θ as well as isolated PKC domains have been described previously [30–33]. Vectors encoding phosphomimetic mutants of PKCδ were generated from the full-length EGFP-tagged PKCδ plasmid using site-directed mutagenesis. A pEGFP-N1 vector was used as GFP-control in experiments. The tags used are expected to not affect protein function [34, 35].

### Cell culture

MCF-7 cells were grown in RPMI 1640 and COS-7 cells were grown in DMEM/High Glucose (Thermo Scientific). All media were supplemented with 10 % fetal bovine serum (Biosera), 100 IU/ml penicillin (Thermo Scientific) and 100 μg/ml streptomycin (Thermo Scientific). RPMI medium was additionally supplemented with 1 mM sodium pyruvate (PAA Laboratories). Cells were grown in 10 cm Petri dishes (Falcon) at 37 °C and 5 % CO_2_. When indicated, cells were treated with 16 nM 12-O-tetradecanoylphorbol-13-acetate (Sigma) or 2 μM GF109203X.

### Immunoprecipitation

For immunoprecipitation procedures, 2 x 10^6^ cells were seeded in 10 cm Petri dishes. Transfections were performed as described previously [36]. Transfection controls were incubated with growth medium without serum or penicillin-streptomycin. For TPA-stimulation, 16 nM TPA was added to the cells after transfection and 16 h after transfection, cells were collected and lysed. Immunoprecipitations were performed using MACS Separation Columns together with μMACS GST Isolation Kit for GST-tagged proteins and μMACS GFP-Tagged Protein Isolation Kit for GFP-tagged proteins (Miltenyi Biotec). For PKCδ-immunoprecipitations, 1 μg anti-PKCδ antibody (Santa Cruz) was used together with MultiMACS protein G kit and μMACS Protein G Microbeads (Miltenyi Biotec) with 1 μg normal rabbit IgG-antibody (Santa Cruz) as control. All immunoprecipitations were performed as described in manufacturer’s protocol with the exception of GST-immunoprecipitations where binding reactions were incubated with beads for 90 min. For all Western blots performed with samples from immunoprecipitation, 2 % of each sample was loaded for input fractions and 48 % was loaded for IP fractions.

### In vitro interaction of recombinant, purified PKCδ and Smac

The in vitro interaction was studied by incubating purified, His-tagged Smac protein (0.5 μg) with GST-tagged PKCδ (0.5 μg, Enzo Life Sciences) in GST-pulldown buffer containing 20 mM Tris-HCl pH 7.5, 0.1 mM EDTA, 100 mM NaCl, 1 mM DTT and 40 μl/ml Complete Protease Inhibitors (Roche). The purified His-Smac had previously been produced in *E. coli* using a pET-28a expression vector. For controls, GST-tagged PKCδ was substituted for purified GST. Immunoprecipitation was thereafter performed as described in the material and methods section.

### Western blot

Western blot was performed as described in a previous publication [37]. Primary antibodies used were anti-PKCδ (1:500, Santa Cruz), anti-Smac (1:500, Santa Cruz), anti-Actin (1:2000, MP Biomedicals), anti-HSV (1:1000, Novagen), anti-GST (1:2000, GE Healthcare) and anti-GFP (1:1000, Invitrogen). Secondary horseradish peroxidase-labeled antibodies used were from GE Healthcare and Dako. For the chemiluminescent reaction, Supersignal Substrate (Thermo Scientific) was used according to manufacturer’s instructions. Chemiluminescence was detected with a LAS-1000 charge-coupled device camera (Fujifilm) and Image Reader LAS-1000 Pro v2.6 software (Fujifilm). Image quantifications were performed using ImageJ 1.48v and by normalizing band intensities to input fractions and control.

### Site-directed mutagenesis

Site-directed mutagenesis was performed using QuikChange II Site-Directed Mutagenesis Kit (Agilent) according to manufacturer’s protocol. 20 μg of FL-PKCδ-EGFP plasmid was used for each PCR-reaction. Primer sequences used for the PCR-reaction were the following: Y64D mutation forward primer – TTCGATGCCCACATCGATGAGGGGCGCGTCATC, Y64D mutation reverse primer – GATGACGCGCCCCTCATCGATGTGGGCATCGAA, Y155D mutation forward primer – CAGGCCAAAATCCACGACATCAAGAACCATGAG, Y155D mutation reverse primer – CTCATGGTTCTTGATGTCGTGGATTTTGGCCTG, Y313D mutation forward primer – GAGCCTGTTGGGATAGATCAGGGTTTCGAGAAG, Y313D mutation reverse primer – CTTCTCGAAACCCTGATCTATCCCAACAGGCTC. All primers were ordered from Invitrogen. Bacteria were grown for 24 h before Miniprep was performed using the JETquick Plasmid Miniprep Spin Kit (Genomed) according to manufacturer’s protocol. The resulting minipreps were checked for successful mutation by sequencing of the plasmids. The minipreps which had incorporated the mutation were then amplified by transformation of XL-2 Blue Ultracompetent cells (Agilent) followed by Maxiprep using the JETstar Plasmid Purification MAXI kit (Genomed) according to manufacturer’s instructions.

### Statistical analysis

All statistical analyses were performed using IBM SPSS Statistics 22. Significance of difference was tested using analysis of variance (ANOVA) followed by Tukey’s HSD test. Differences were considered significant if the *p*-value was below 0.05.

## Abbreviations

cIAP: cellular inhibitor of apoptosis protein
EGFP: enhanced green fluorescent protein
GFP: green fluorescent protein
GST: glutathione S-transferase
HSV: herpes simplex virus
MTS: mitochondrial targeting signal
PKC: protein kinase C
TNF: tumor necrosis factor
TPA: 12-O-tetradecanoylphorbol-13-acetate
XIAP: X-linked inhibitor of apoptosis protein
